# Parent Attitudes about Childhood Vaccines: Point Prevalence Survey of Vaccine Hesitancy in an Irish Population

**DOI:** 10.3390/pharmacy9040188

**Published:** 2021-11-23

**Authors:** Sarah Marshall, Anne C. Moore, Laura J. Sahm, Aoife Fleming

**Affiliations:** 1 Pharmaceutical Care Research Group, School of Pharmacy, University College Cork, T12 YN60 Cork, Ireland; smarshall5786@gmail.com (S.M.); L.Sahm@ucc.ie (L.J.S.); 2 School of Biochemistry and Cell Biology, University College Cork, T12 XF62 Cork, Ireland; anne.moore@ucc.ie; 3 Pharmacy Department, Mercy University Hospital, T12 WE28 Cork, Ireland

**Keywords:** vaccination, vaccine hesitancy, PACV, parents’ beliefs, immunisation

## Abstract

Understanding parental attitudes to their children’s vaccination is critical to developing and implementing interventions that address parents’ hesitancy and improve vaccine uptake. The Parent Attitudes about Childhood Vaccines (PACV) survey is a validated tool for identifying vaccine hesitancy in parents. We evaluated the rate of vaccine hesitancy and areas of concern regarding childhood vaccinations using an adapted version of the PACV survey, in a convenience sample of parents attending a STEM (Science, Technology, Engineering and Mathematics) outreach event in Ireland, in 2018. A score ≥ 50 identified vaccine hesitant parents. Of 105 parents who completed the survey, the prevalence of vaccine hesitancy was 6.7%, (7/105). Parents had concerns around vaccine side effects (36.2%, *n* = 38), vaccine safety (20%, *n* = 21) and the number of vaccines administered (13.3%, *n* = 14). Parents trusted the vaccine information they received (85.6%, *n* = 90) and 81.9% (*n* = 86) believed that the vaccine schedule was good for their child. The findings indicate the presence of vaccine hesitancy in parents in Ireland regarding paediatric vaccines with further research necessary to address parents’ vaccine concerns. Future research should explore further, by qualitative methods, parents’ vaccine concerns. There is also potential to identify vaccine hesitant parents with the PACV survey as a surveillance method in healthcare settings; for example, in community pharmacies, family doctor clinics and out-patient clinics.

## 1. Introduction

Prophylactic vaccination of infants and children with several vaccines is the cornerstone of effective immunisation programmes against a variety of childhood diseases. Despite these benefits, immunisation rates in many countries including Ireland, and for some vaccines, remain suboptimal in spite of vaccine availability [1]. This has led to a series of potentially preventable disease (e.g., measles, pertussis) outbreaks internationally [2,3]. Mandatory childhood vaccination programmes have led to increased vaccine coverage in countries such as Italy, France and Germany [4]. Ireland does not have a mandatory vaccination programme, and vaccination coverage is not optimal for some vaccines. Ireland reported 2762 cases of mumps in 2019, an almost five-fold increase on 2018, with five measles outbreaks reported in 2019 also [5]. Waning vaccine confidence has taken a toll on immunisation programmes globally [6]. While this reduction in confidence is multifactorial, a key contributory factor is vaccine hesitancy, recognised by the World Health Organisation as one of the greatest threats to global health in 2019 [7]. Vaccine hesitancy refers to a delay in acceptance or refusal of vaccines despite availability of vaccine services, is complex and context specific varying across time, place, and vaccines, and is influenced by factors such as complacency, convenience and confidence [8]. This definition depolarises the pro- or anti-vaccine stance. In reality, vaccine hesitant individuals are a heterogeneous group in a continuum, ranging from total acceptance to complete refusal [6]. It is known that vaccine hesitancy is highly variable and context specific, varying across time, place and the specific vaccine involved [8,9]. It is a multi-layered phenomenon, related to prior beliefs about vaccines [10], perceived benefits of vaccines, attitudes towards vaccines [11], previous experiences with vaccines [12], socioeconomic status [13], number of children [14], and marital status [13]. Given this heterogeneity, identifying vaccine hesitant individuals in need of guidance is challenging. The Parent Attitudes about Childhood Vaccines (PACV) survey was developed to begin to address this challenge [15]. It is a self-administered instrument and contains 15 items under three domains: behaviour, vaccine safety and efficacy, and general vaccine attitudes [15]. The PACV survey has been validated to identify vaccine hesitant parents and to predict future vaccine refusal [16,17]. More recently, the survey has since been translated and tested in multiple languages [18,19,20], a short scale has been developed [21], and the instrument has been used in a wide variety of contexts [22]. However, due to the context specificity of vaccine hesitancy, the reliability of the PACV survey should be assessed in different geographic areas and demographic samples of parents [17]. Therefore, the aim of this study was to use the PACV to measure the prevalence of vaccine hesitancy in a population of parents attending a community science outreach event in Cork, Ireland.

## 2. Materials and Methods

This study was conducted on the 18 November 2018 at the “Community Festival of Science”, the finale of Cork Science Festival, involving highly interactive exhibitions and demonstrations. Cork Science Festival is a main partner of Science Week Ireland, one of the largest Irish STEM (Science, Technology, Engineering and Mathematics) engagement events. This event was chosen for recruitment as it was promoted as a family event with parents and children of all ages encouraged to attend; in this way, direct recruitment of parents was possible. Any parent (over the age of 20 years) attending the event was eligible for participation in the study. Attendees were approached on an ad hoc basis throughout the event and invited to read the participant information leaflet about the study, to provide written informed consent, and to complete the PACV survey. The survey was adapted for use in the Irish context e.g., highest education level reached (first, second, third or fourth level options in line with Irish context), and the replacement of the term “shot” in the survey questions with “vaccine”. In addition, questions pertaining to marital status, ethnicity and household income were removed as these were not directly relevant to the calculation of the hesitancy score and may have negatively impacted recruitment. The Irish childhood immunisation schedule is provided as Supplementary information (Table S1).

Each of the 15 PACV survey items was scored, in accordance with the original PACV system: hesitant responses are assigned a 2, ‘don’t know or not sure’ a 1, and non-hesitant responses a 0. Item scores were summed in an unweighted fashion to obtain a total raw score. The total raw score was then converted to a scale ranging from 0 (least hesitant) to 100 (most hesitant), using simple linear transformation, with a score ≥ 50 that identified vaccine hesitant parents, while a score < 50 that identified non-hesitant parents [15]. Continuous variables were described by medians and IQRs. Categorical variables were described by counts and percentages. Associations between categorical variables were investigated using Fisher’s exact test. *p* values of < 0.05 were considered statistically significant.

## 3. Results

A total of 105 parents participated in the study, Table 1 outlines the participants demographic details.

The survey was self-administered in less than five minutes and no issues were reported by participants with its completion. All 105 participants answered all questions. The most commonly reported age range was 40–49 years (*n* = 57, 54.3%). The education level of most respondents (*n* = 79, 75.2%) was third level (higher education in universities, institutes of technology and other colleges of education). The majority of respondents (*n* = 53, 50.5%) had two children.

**Figure 1:**
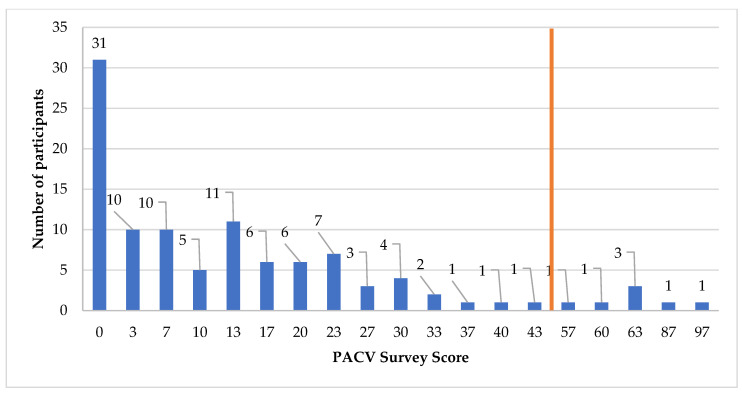
Participant Parent Attitudes about Childhood Vaccines (PACV) scores, with reference line at score 50.

**Figure 2:**
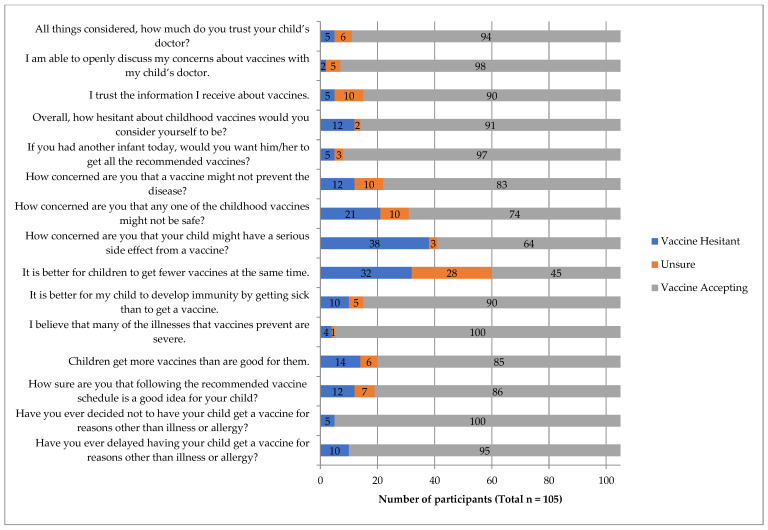
Responses to individual PACV survey items.

Overall, 6.7% (*n* = 7) of participants were identified as vaccine hesitant, with a converted score ≥ 50 (Figure 1). The median (IQR) converted score was 10 (0, 20), (mean score of 20.59, 95% CI 16.12 to 25.06). There were no statistically significant differences between the vaccine hesitant and non-hesitant groups based on age (*p* = 1.000), education (*p* = 0.182) or number of children (*p* = 1.000). A summary infographic of individual survey items and results depicting vaccine hesitancy is provided in Figure 2. The highest number of hesitant responses was associated with the survey item “How concerned are you that your child might have a serious side effect from a vaccine?” with 36.2% of participants (*n* = 38) indicating they were somewhat concerned or very concerned (Figure 2). Conversely, the lowest number of hesitant responses was associated with survey item “I am able to openly discuss my concerns about vaccines with my child’s doctor”, with only 1.9% of participants (*n* = 2) reporting concern (Figure 2). In response to the item “If you had another infant today, would you want him/her to get all the recommended vaccines?”, 4.7% (*n* = 5) participants answered “No”, while 2.9% (*n* = 3) answered “Don’t know”; seven of these eight participants were identified as vaccine hesitant when scoring was complete. Most parents reported a non-hesitant belief that many of the diseases that vaccines prevent are severe (95.2%, *n* = 100) and were accepting of vaccines in that they trust in vaccine information (85.6%, *n* = 90).

In this study, the seven identified vaccine hesitant participants indicated that they would not, or were unsure whether they would, consent to vaccination for future infants in their care.

## 4. Discussion

The aim of this study was to measure the prevalence of paediatric vaccine hesitancy using the PACV survey, in a population of parents attending a STEM outreach event. The prevalence of vaccine hesitancy among participants was relatively low, at 6.7%. Other prevalence studies which have used the PACV survey to determine vaccine hesitancy found rates of 7.7% in Italy [23], 11.6% in Malaysia [18], 15% in Canada [24], and 26% in the United States of America [25]. In a recent study in Ireland in a clinical setting, a PACV determined vaccine hesitancy rate of 14.4% was found [26]. The reported prevalence of vaccine hesitancy has been highly variable, ranging from 5.9% in a population of mothers in Washington State [27], to 26% of parents attending a paediatric emergency department of a tertiary hospital in Seattle [25]. In our study, the issues parents were mostly concerned about were vaccine side effects and the number of vaccines on the childhood schedule. We found that 36.2% of all participants reported concerns of side effects, but only 18.5% of these were identified as vaccine hesitant (according to the PACV survey), and 81.6% of these indicated that they would consent to vaccination for future infants.

This point prevalence study did not permit long term follow-up of future immunisation practices and it is known that intention alone does not necessarily predict future vaccine uptake; a disparity known as the intention–behaviour gap [28]. However, this study suggests that the PACV survey may be used as a rapid screening tool to identify potentially vaccine hesitant parents as candidates for more intensive targeted vaccine education and decision support. We found that 94.3% of all participants (*n* = 99) agreed with the statement “I am able to openly discuss my concerns about vaccines with my child’s doctor”, and on a Likert scale ranging from 0 (do not trust at all) to 10 (completely trust), 89.5% of participants (*n* = 94) reported a score of ≥ 8 in response to the item “All things considered, how much do you trust your child’s doctor?” This aligns with data collected in the Wellcome Trust’s Global Health Monitor in Ireland during this time, where 93% of Irish participants reported that they trusted these healthcare providers (HCPs), and 85% trusted them most for medical and/or health advice [29].

In this study, 30.5% of participants felt that it is better for children to get fewer vaccines at the same time. Similar to side effect concern, 78.1% of these participants declared they would vaccinate future children, and none were identified as vaccine hesitant. This suggests that while parents have concerns regarding the number of childhood vaccines or vaccine side effects, they may still choose to vaccinate in spite of these concerns. This hypothesis was reported by Whelan et al. in an Irish study of 564 parents where they found a PACV hesitancy rate of 14.4%, which was three times higher than the reported non-vaccination rate [26]. Whelan et al. reported a higher hesitancy rate in a parent population attending a hospital paediatric clinic than our study (6.7%); this may be as a result of our study population attending a STEM event which may suggest a lower level of mistrust or hesitancy regarding vaccines. Both studies were conducted in 2018, with similar levels of education status reported. Further information regarding other demographics or health status of the children was not available to derive any further comparison in the different hesitancy rates. This highlights the importance of using the PACV as a vaccine hesitancy surveillance tool in different geographical regions and parent demographic groups, to explore the impact of context on vaccine hesitancy [17].

Factors such as number of children [14] and level of education [6] have been identified as determinants of vaccination. However, no such associations were identified in this study population. Fear of vaccine side effects has been consistently identified as a driver of vaccine hesitancy [30]. This fear is often associated with new vaccines [31], novel delivery systems [32], or the sensationalist dissemination of vaccine misinformation. Vaccine hesitancy may also be driven by the belief that too many vaccines can “overwhelm the immune system” [33]. In Ireland, the primary childhood immunisation schedule and school programme involve the administration of vaccines on seven occasions from birth to approximately 13 years, and includes both single and combination vaccines to protect against 13 diseases (Supplementary Table S1).

This study has found that, in an Irish parent population, it appears that parents will consent to vaccination in spite of concerns regarding vaccine safety and the number of vaccinations on the schedule. Moving forward, allaying these concerns is important in preventing their escalation, especially in the face of increased access to reliable, evidence-based information sources [34]. The PACV survey was found to be a quick and effective means to identify vaccine hesitant parents and should be considered as a screening tool by HCPs in practice. The role of HCPs such as community pharmacists, family doctors and public health nurses in guiding vaccine decisions has been identified in research conducted elsewhere [14,35]. Effective interactions with HCPs can alleviate the concerns of vaccine supportive parents and can motivate a vaccine hesitant parent towards acceptance [35]. It has been shown that parents prefer strong, unambiguous recommendations from their HCP [31]. Development of continuous professional development (CPD) programmes to provide HCPs with information to adequately address vaccine concerns, and communication strategies to support HCPs in recommending vaccines with confidence would support efforts in this area. The importance of communication in addressing vaccine hesitancy has been identified [36], and future initiatives to guide HCPs interactions with parents regarding vaccines could address these concerns by means of a nationally implemented programme [37].

A strength of this study is the use of a previously validated survey instrument (the PACV) to determine vaccine hesitancy in parents. A potential limitation of this study is the recruitment of participants from a science outreach event which is educational in nature, which may have increased the potential for increased vaccine acceptance. However, the vaccine hesitant parents had varying levels of education which may help to mitigate this limitation. It has been shown in previous studies that education level does not predict vaccine hesitancy [6]. In addition, there is the potential that self-selection bias impacted upon the results; those who are vaccine hesitant may have been less likely to participate in the survey upon invitation at the event. Conversely, the collection of data in an anonymous manner minimises the potential for social desirability bias. The study has a small sample size which limits the generalisability of the findings to the wider Irish population.

## 5. Conclusions

The PACV survey was efficiently self-administered in a population of parents attending a STEM event. The prevalence of vaccine hesitancy was 6.7%. The survey results found that most parents reported as being non-hesitant on many areas such as trust in vaccines information and in the vaccination schedule. Some concern was expressed in the domain of vaccine safety and side effects. There is potential for the PACV survey to be used by HCPs such as pharmacists and family doctors to rapidly identify those parents, and their beliefs or concerns, in need of additional vaccine decision support and communication.

## Figures and Tables

**Table 1:** Participant demographics (*n* = 105 participants).

Demographic Information	Values (%)
Age range (years):	
20–29	2 (1.9%)
30–39	40 (38.1%)
40–49	57 (54.3%)
50–59	2 (1.9%)
60–69	4 (3.8%)
Number of children:	
1	21 (20%)
2	53 (50.5%)
3	26 (24.8%)
4 or more	5 (4.8%)
Level of education:	
First	1 (1%)
Second	11 (10.5%)
Third	79 (75.2%)
Fourth	14 (13.3%)

## Data Availability

Not applicable.

## Supplementary Materials

The following are available online at https://www.mdpi.com/article/10.3390/pharmacy9040188/s1, Table S1: Routine paediatric vaccination schedule in Ireland (November 2018).
