# Supplementary material for: Parent Attitudes about Childhood Vaccines: Point Prevalence Survey of Vaccine Hesitancy in an Irish Population
Source: Pharmacy (Basel). 2021 Nov 23;9(4):188. doi: 10.3390/pharmacy9040188 (PMC8628985; doi:10.3390/pharmacy9040188)
Supplement: Supplementary file 1 [file pharmacy-09-00188-s001.zip › pharmacy-1469150-SI.pdf]

Supplementary information:

Supplementary Table S1: Routine paediatric vaccination schedule in Ireland (Nov 2018).

**Vaccine**	**Schedule**
Primary immunisation schedule:	
6 in 1, PCV, MenB, Rotavirus	2 months
6 in 1, MenB, Rotavirus	4 months
6 in 1, PCV, MenC	6 months
Measles Mumps Rubella (MMR), MenB	12 months
Hib/MenC, PCV	13 months
School Programme:	
4 in 1, MMR	Junior infants primary school (age 5-6 years)
Human Papillomavirus (for girls in 2018), Tdap (tetanus, diphtheria, pertussis), Meningococcal ACWY	First year secondary school (age 12-13)

6 in 1: Diphtheria, Tetanus, (Pertussis), Hib (Haemophilus influenzae type b), Polio (inactivated), Hepatitis B.

PCV: Pneumococcal conjugate vaccine

MenB: Meningococcal B vaccine

MenC: Meningococcal C vaccine

Hib/MenC: Haemophilus influenzae type b, Meningococcal type b combined.

4 in 1: Diphtheria, polio, tetanus, pertussis.

Reference: https://www.hse.ie/eng/health/immunisation/pubinfo/pcischedule/immschedule/
